# Reply to: Noninvasive positive pressure ventilation after extubation: features and outcomes in clinical practice

**DOI:** 10.5935/0103-507X.20160017

**Published:** 2016

**Authors:** Liria Yuri Yamauchi, Maise Figueiroa, Leda Tomiko Yamada da Silveira, Teresa Cristina Francischetto Travaglia, Sidnei Bernardes, Carolina Fu

**Affiliations:** Department of Human Movement Sciences, Universidade Federal de São Paulo - Santos (SP), Brazil.; Department of Physiotherapy, Communication Sciences & Disorders, and Occupational Therapy, Faculdade de Medicina, Universidade de São Paulo - São Paulo (SP), Brazil.

The authors gratefully acknowledge the comments made by the researchers. We will analyze the methodologies currently used by our group and the cited studies to understand the differences described in the letter. Additionally, we would like to emphasize that the study was a prospective cohort study, in which the researchers collected data on a daily basis from the hospital specialty units included in the study. The hospital where the study was conducted is part of the largest hospital complex in Latin America. It is a high complexity tertiary care hospital located in Sao Paulo city. This factor contributed to the heterogeneity of the study population because many units with different specialties were included.

In our study,^(1)^ the parameters of noninvasive ventilation (NIV) that were evaluated were collected on the last day of NIV use, i.e., the day of NIV withdrawal due to either success or failure. The parameters at the time of collection were recorded and compared.

In the impressive clinical trial reported by Esteban et al.,^(2)^ NIV was applied upon detection of acute respiratory failure, and the parameters used were adjusted according to the tidal volume and expected respiratory rate. We believe that in a clinical trial, the parameters are more controlled and that the methodology of that trial did not detail when and how the inspiratory pressure parameters were collected.

In the study by Rana et al.^(3)^ the parameters were collected four times daily, and their median values were analyzed. In contrast, our study evaluated the parameters collected on the last day of NIV use. Another important distinction of our study was the population. Rana et al.^(3)^ studied a very specific population that had severe disease (acute lung injury and acute respiratory distress syndrome).

Regarding the study by José et al.,^(4)^ the authors reported contrasting results to those published by Esteban et al.^(2)^ However, the study designs were very different because José et al.^(4)^ performed a prospective study with no control group and no randomization, which are factors that weakened their results. We consider the results obtained in clinical practice to be of greater importance. We believe that from these data, we can plan new controlled and randomized clinical trials to further evaluate the hypothesis.

We will try to strengthen the hypothesis in future studies by carefully evaluating the parameters of inspiratory positive airway pressure and expiratory positive airway pressure to identify possible markers for detecting the interruption of NIV in cases of failure.

We appreciate the suggestion to expand the study. However, cohort studies are usually very expensive, and the costs for implementing such studies, given the characteristics and size of our country, would make fulfilling this suggestion challenging. In our country, many issues must be considered, such as whether the hospital is public or private, is a teaching or community hospital, or is located in a large city or smaller town. The Latin American Institute for Sepsis presented data that clearly illustrated the differences related to the availability of intensive care units (ICU) beds in Brazil with respect to the comparison between public and private hospitals. Access to the ICUs of public hospitals is much more restricted than access to the ICUs of private hospitals.^(5)^ These peculiarities would make studies that involve many institutions have heterogeneous groups, especially in the case of non-interventional studies.

We agree that a more homogeneous study population may lead to clearer information regarding the applied therapies. Our study was limited due to a heterogeneous population, although as shown in table 1 of our article,^(1)^ the study groups presented a homogeneous distribution as to the causes of respiratory failure. We would like to reiterate that the purposes of our study were to evaluate the use of NIV after extubation in a large university hospital in Brazil, to estimate the rates of NIV success and failure and to consider the possible factors associated with failure.

Undoubtedly, new randomized controlled clinical trials may further elucidate the relationship between NIV parameters and patient outcomes.

We have considered all of the aforementioned limitations, and we appreciate the comments of the researchers. NIV has emerged as very promising technique that can significantly reduce mortality in various types of acute respiratory failure when applied early.^(6)^ We believe it is crucial to continue research studies of all aspects of NIV.

Once again, we are grateful for the comments and hope that we have addressed the points that were raised.

Liria Yuri Yamauchi Department of Human Movement Sciences,
Universidade Federal de São Paulo - Santos (SP), Brazil.Maise Figueiroa, Leda Tomiko Yamada da Silveira, Teresa Cristina Francischetto
Travaglia, Sidnei Bernardes and Carolina Fu Department of Physiotherapy,
Communication Sciences & Disorders, and Occupational Therapy, Faculdade de
Medicina, Universidade de São Paulo - São Paulo (SP),
Brazil.
